# TLR-mediated albuminuria needs TNFα-mediated cooperativity between TLRs present in hematopoietic tissues and CD80 present on non-hematopoietic tissues in mice

**DOI:** 10.1242/dmm.023440

**Published:** 2016-06-01

**Authors:** Nidhi Jain, Bhavya Khullar, Neelam Oswal, Balaji Banoth, Prashant Joshi, Balachandran Ravindran, Subrat Panda, Soumen Basak, Anna George, Satyajit Rath, Vineeta Bal, Shailaja Sopory

**Affiliations:** 1 National Institute of Immunology, New Delhi 110067, India; 2 Pediatric Biology Center, Translational Health Sciences and Technology Institute, Faridabad 121001, National Capital Region, India; 3 Department of Pathology, All India Institute of Medical Sciences, New Delhi, India; 4 Institute of Life Sciences, Bhubaneswar, India

**Keywords:** CD80, IL-10, Proteinuria, TLR, TNFα

## Abstract

Transient albuminuria induced by pathogen-associated molecular patterns (PAMPs) in mice through engagement of Toll-like receptors (TLRs) is widely studied as a partial model for some forms of human nephrotic syndrome (NS). In addition to TLRs, CD80 has been shown to be essential for PAMP-mediated albuminuria. However, the mechanistic relationships between TLRs, CD80 and albuminuria remain unclear. Here, we show that albuminuria and CD80-uria induced in mice by many TLR ligands are dependent on the expression of TLRs and their downstream signalling intermediate MyD88 exclusively in hematopoietic cells and, conversely, on CD80 expression exclusively in non-hematopoietic cells. TNFα is crucial for TLR-mediated albuminuria and CD80-uria, and induces CD80 expression in cultured renal podocytes. IL-10 from hematopoietic cells ameliorates TNFα production, albuminuria and CD80-uria but does not prevent TNFα-mediated induction of podocyte CD80 expression. Chitohexaose, a small molecule originally of parasite origin, mediates TLR4-dependent anti-inflammatory responses, and blocks TLR-mediated albuminuria and CD80-uria through IL-10. Thus, TNFα is a prominent mediator of renal CD80 induction and resultant albuminuria in this model, and small molecules modulating TLR-mediated inflammatory activation might have contributory or adjunct therapeutic potential in some contexts of NS development.

## Abstract (precis)

**Summary:** Systemic TNFα mediates myeloid cell and podocyte cross-talk to cause LPS-induced mouse microalbuminuria, a partial model of human nephrotic syndrome, pointing to potential adjunct therapeutic approaches.

## INTRODUCTION

Minimal change disease nephrotic syndrome (NS) is the commonest form of NS in the paediatric age group (Bagga and Mantan, 2005; Eddy and Symons, 2003). It is largely sensitive to corticosteroids (Indian Society of Pediatric Nephrology et al., 2009), suggesting an inflammatory aetiopathogenetic component. The molecular origins and effector pathways mediating such an inflammatory mechanism for minimal change NS remain unclear despite a number of reports identifying potential candidates (Sahali et al., 2014; Shimada et al., 2011), especially cytokines such as interleukin 1 (IL-1) (Bricio et al., 1992), IL-8 (Cho et al., 2003), IL-13 (Abdel-Hafez et al., 2009; Lai et al., 2007) and TNFα (Raveh et al., 2004; Suranyi et al., 1993). Many forms of systemic inflammation, including sepsis, are associated with proteinuria (Drumheller et al., 2012; Emara et al., 2013; Schreiber et al., 2012) with high levels of pro-inflammatory cytokines such as IL-1, IL-6 and TNFα (Hotoura et al., 2011; Makhija et al., 2005; Martin et al., 1994), and some cases of NS have been shown to be responsive to TNFα blockade (Raveh et al., 2004). Activation of macrophages through classic pathways provides a major source of pro-inflammatory cytokines, and regulation of classic activation, such as through alternative activation, can lead to amelioration of inflammation and sepsis (Feng et al., 2014; Martinez and Gordon, 2014; Panda et al., 2012; Zhou et al., 2014).

Animal models of NS have been useful in identifying various possible components of the immune-based pathology. Genetic approaches have been used in mouse models to determine the roles of angiopoetin-like-4 (Clement et al., 2011), c-mip (Zhang et al., 2010) and nephrin (Eremina et al., 2002), among others. Use of plasma from affected individuals (McCarthy et al., 2010), lipopolysaccharide (LPS), puromycin aminonucleoside (PAN) (Muroya et al., 2012) and doxorubicin (Artunc et al., 2008) have also been reported as inducing some NS-like disease components in mice. LPS-mediated transient albuminuria is accompanied by some renal alterations that are similar to those in human NS (Clement et al., 2011; Wei et al., 2008) along with an inflammatory response, and LPS-induced albuminuria is one of the more widely used NS models. Other inflammatory agonists of members of the Toll-like receptor (TLR) family have also been shown to induce similar disease, and blockade of type I interferon signalling can prevent LPS-mediated albuminuria (Gurkan et al., 2013). CD80, an immune co-stimulatory molecule commonly expressed in bone marrow (BM)-derived hematopoietic cell lineages, is found excreted in the urine of NS individuals (Garin et al., 2009) and has been shown to be essential for disease in mouse models of albuminuria (Ishimoto et al., 2011; Reiser et al., 2004). Many TLRs are expressed by renal tissue cells, such as glomerular podocytes (Clement et al., 2011; Ishimoto et al., 2013a; Shimada et al., 2012), and TLR ligands as well as sera from NS-affected individuals induce CD80 expression on podocytes *in vitro* (Chang et al., 2013; Ishimoto et al., 2013b). Thus, although MCD has been proposed to be an outcome of ‘two hits’, with TLRs and CD80 as the two major contributors to the pathology (Shimada et al., 2011), and although many components of signalling intermediates mediating podocyte changes have been described (Greka and Mundel, 2012; Hattori et al., 2011; Reiser et al., 2014), the precise relationships between TLR expression, CD80 expression and albuminuria are still unclear.

Given these data, we show evidence that the most likely mechanistic model for TLR-mediated mouse albuminuria involves systemic activation of myeloid cells followed by TNFα-mediated induction of CD80-dependent renal tissue dysfunction. Our results also indicate that not only TNFα antagonism but also small molecules inducing alternative activation of macrophages block TLR-induced albuminuria, opening up possibilities for new targets for adjunct therapeutic intervention in some contexts of human NS.

## RESULTS

### *CD80*^−/−^ mice are resistant to TLR-mediated albuminuria

**Fig. 1.**
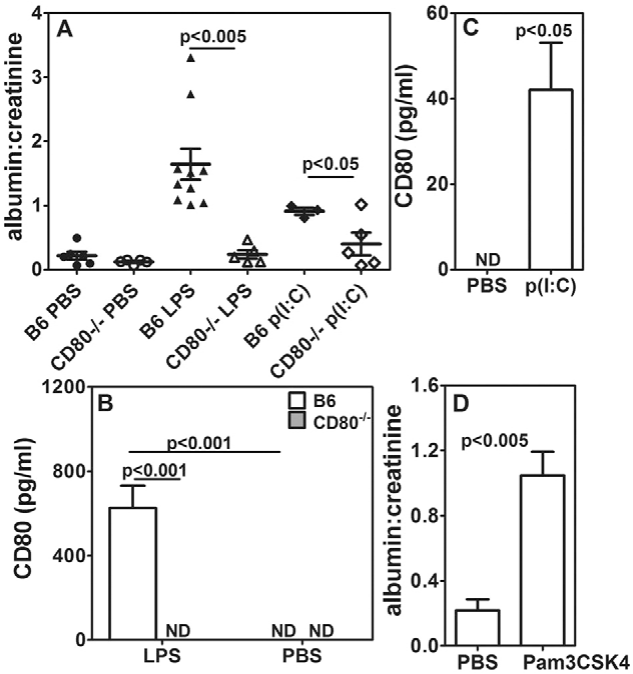
***CD80***^−/−^ **mice are resistant to LPS- and poly(I:C)-mediated albuminuria.** (A) Comparison of urinary albumin:creatinine ratios between B6 and *CD80*^−/−^ mice given PBS, LPS or poly(I:C) [number of mice per group as follows: B6+PBS=6, B6+LPS=10, B6+p(I:C)=3; *CD80*^−/−^=5 in all groups]. (B) Comparison of urinary CD80 levels between B6 and *CD80^−/−^* mice given PBS or p(I:C) (five mice per group). (D) Levels of albumin in urine of B6 mice given PBS or Pam3CSK4 (six mice per group). The results represent mean±s.e.m. ND, not detectable.

*CD80*^−/−^ mice have been shown to be resistant to LPS-induced albuminuria (Reiser et al., 2004). We extended these findings to see whether they are resistant to other TLR ligands as well. Wild-type C57BL/6 (B6) and *CD80*^−/−^ mice were given optimized (100 µg/mouse, intraperitoneally) doses of the TLR4 ligand LPS or the TLR3 ligand polyinosinic:polycytidylic acid [poly(I:C)], and urine was collected 24 h later from individual mice to measure albumin, creatinine and CD80 levels. Although B6 mice showed an elevated albumin:creatinine ratio in urine in response to TLR4 or TLR3 ligation, *CD80*^−/−^ mice showed no significant change (Fig. 1A). CD80 was detectable in the urine of B6 mice that had been given LPS, but not in its absence (Fig. 1B). CD80-uria in B6 mice was also detected in response to poly(I:C) (Fig. 1C). We also tested Pam3CSK4, a synthetic TLR2 ligand, in B6 mice for its ability to induce albuminuria because, in lupus-prone mice, it is reported to induce albuminuria (Pawar et al., 2009). Similar to ligation of TLR3 and TLR4, TLR2 ligation also resulted in an enhanced albumin:creatinine ratio (Fig. 1D).

### CD80 expression on BM-derived cells is dispensable for induction of albuminuria

**Fig. 2.**
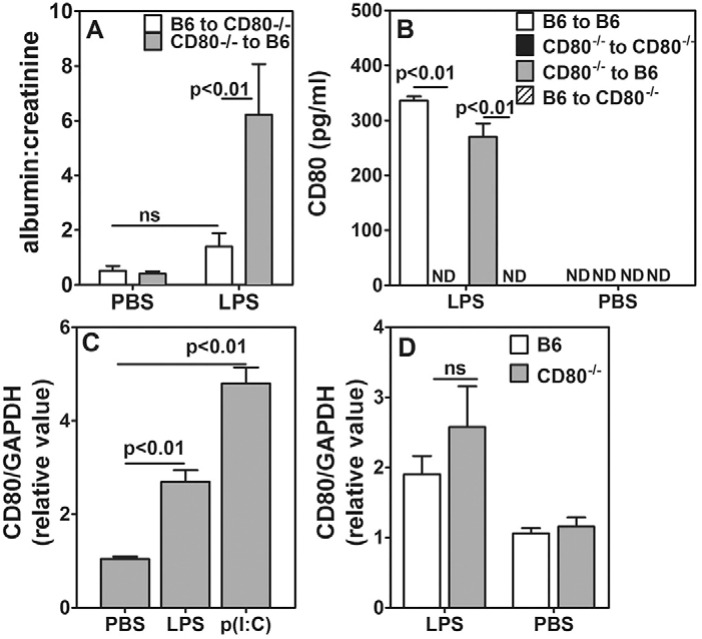
**CD80 expression on podocytes is essential for albuminuria.** (A) Comparison of urinary albumin:creatinine ratios in BM chimeric mice given LPS or PBS. The key identifies BM donors and recipients (five mice per group). (B) CD80 levels in BM chimeric mice given LPS or PBS (five mice per group). ‘B6’ in panels A and B indicates B6.SJL. (C) Relative *CD80* mRNA expression in E11 podocytes exposed to serum from B6 mice that had been treated as indicated (*n*=6, data from three independent experiments). (D) Relative *CD80* mRNA expression in E11 podocytes exposed to serum from B6 and *CD80*^−/−^ mice given LPS or PBS (*n*=6, data from three independent experiments). The results represent mean±s.e.m. ND, not detectable; ns, not significant.

CD80 is expressed normally by many BM-derived cell lineages. Although renal cells are not known to express CD80 under physiological conditions, they do express it under various stress conditions, such as upon exposure to TLR3 and TLR4 ligands or chronic hypoxia (Chang et al., 2013; Shimada et al., 2012). We also observed expression of CD80 in the podocytes of B6 mice that had been treated with LPS, but not in untreated mice (Fig. S1). Podocytes from *CD80*^−/−^ mice, as expected, did not show presence of CD80 whether treated with LPS or not (Fig. S1). Neither our data above nor those published previously (Reiser et al., 2004) establish whether the presence of CD80 on renal cells and/or on BM cells is necessary for LPS-mediated albuminuria. To address this, we generated homologous and heterologous BM chimeras, where irradiated *CD80*^−/−^ and B6.SJL mice received BM cells from either B6.SJL or *CD80*^−/−^ mice (i.e. B6.SJL to B6.SJL, *CD80*^−/−^ to *CD80*^−/−^, B6.SJL to *CD80*^−/−^ and *CD80*^−/−^ to B6.SJL combinations). At 4-6 weeks after transfer, we tested for chimerism in these mice. Representative data from blood analyses of B6.SJL-to-*CD80*^−/−^ and *CD80*^−/−^-to-B6.SJL mice show that chimerism was established successfully (Fig. S2A). Further, we stained splenic cells after red blood cell lysis in order to examine whether myeloid cells in the spleen can express CD80 after LPS injection. Myeloid cells, primarily comprising macrophages from B6.SJL mice (CD45.1^+^ CD11b^+^) showed CD80 staining (Fig. S2B), whereas those from *CD80*^−/−^ mice (CD45.2^+^ CD11b^+^) did not (Fig. S2C). Splenic macrophages from *CD80*^−/−^-to-B6.SJL chimeric mice (CD11b^+^) did not stain for CD80 (Fig. S2D). Sera from *CD80*^−/−^-to-B6.SJL chimeric mice also did not show elevation of serum CD80 levels in response to LPS (Fig. S2E), further confirming appropriate reconstitution following lethal radiation. For albuminuria experiments, LPS was given 10-12 weeks post chimerization. Recipient B6.SJL mice, which received BM from *CD80*-null mice showed a higher albumin:creatinine ratio in response to LPS (Fig. 2A). In contrast, *CD80*^−/−^ recipient mice, which received BM from B6.SJL mice, did not show any significant enhancement in the albumin:creatinine ratio in response to LPS over and above the background (Fig. 2A). Homologous *CD80*^−/−^-to-*CD80*^−/−^ chimeric mice showed no albuminuria, whereas B6.SJL-to-B6.SJL mice showed high levels of albuminuria, as expected (Fig. S3A). When urinary CD80 excretion was evaluated in these mice, *CD80*^−/−^ recipients did not excrete CD80 in urine whether they were reconstituted with B6.SJL or *CD80*^−/−^ BM (Fig. 2B). These data suggest that CD80 expression on radiation-resistant cells such as podocytes is necessary for the induction of albuminuria, whereas CD80 expression on BM-derived cells is not required, and that the radiation-resistant renal cells expressing CD80 excrete it in urine following treatment with LPS.

We examined the induction of CD80 expression in renal cells using the E11 mouse podocyte cell line. Sera were collected from B6 mice that had been treated two hours earlier with LPS or poly(I:C). When these ligands were added to cultured E11 cells, *CD80* mRNA levels were upregulated compared to those in control serum from PBS-treated mice (Fig. 2C). Serum from LPS-treated *CD80*^−/−^ mice also exhibited upregulated *CD80* mRNA levels in E11 cells (Fig. 2D). Thus, B6 and *CD80*^−/−^ mice have similar capabilities to trigger induction of CD80 expression in renal cells, and the absence of albuminuria in *CD80*^−/−^ mice can be attributed to the absence of renal CD80 expression.

### TLR signalling in non-hematopoietic cells is dispensable

The data above still do not clarify the mechanism by which CD80 is upregulated on podocytes in pathophysiological conditions. The ability of serum from LPS-treated *CD80*^−/−^ mice to induce CD80 expression in the podocyte cell line indicates two possibilities. Firstly, the continued presence of LPS in serum might be directly responsible for TLR4 ligation on renal cells, leading in turn to upregulation of CD80. Alternatively, a soluble factor in serum might be responsible for CD80 upregulation on renal cells. To test these possibilities, we critically analysed the role of TLR4 in albuminuria.

**Fig. 3.**
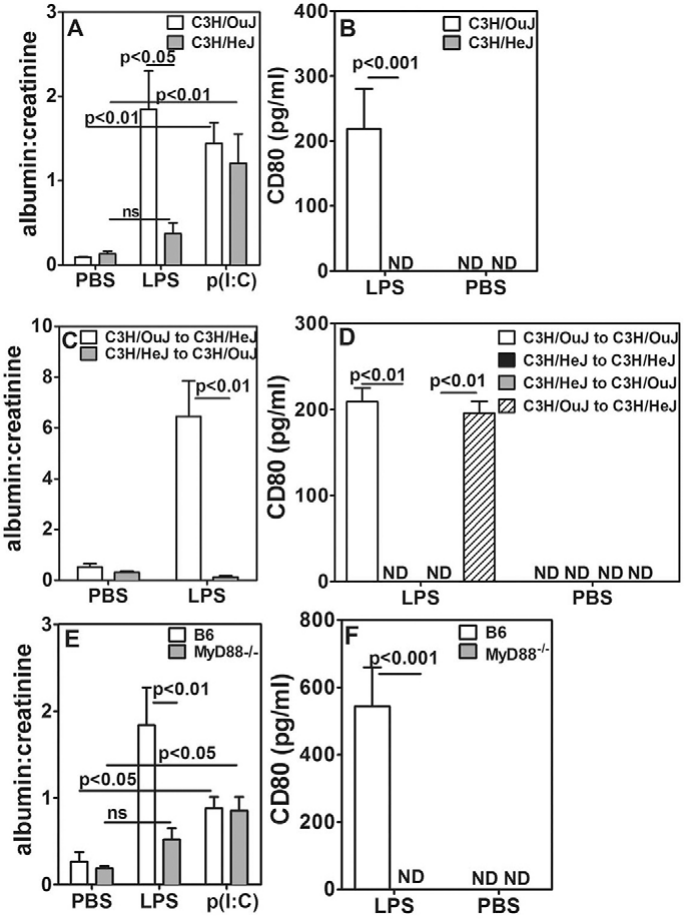
**TLR expression and signalling in non-hematopoietic cells is dispensable for induction of albuminuria.** (A) Comparison of urinary albumin:creatinine ratios in C3H/OuJ and C3H/HeJ mice that had been treated with PBS, LPS or p(I:C) (five mice per group). (B) Comparison of urinary CD80 levels in C3H/OuJ and C3H/HeJ mice given LPS or PBS (eight mice per group). (C) Comparison of urinary albumin:creatinine ratios in BM chimeric mice in response to LPS (five mice per group). (D) CD80 levels in BM chimeric mice given LPS or PBS (five mice per group). (E) Comparison of urinary albumin:creatinine ratios in B6 and *MyD88*^−/−^ mice given LPS, p(I:C) or PBS (five mice per group). (F) Comparison of urinary CD80 levels in B6 and *MyD88*^−/−^ mice given LPS or PBS (eight mice per group). Keys identify BM donors and recipients. Data represents mean±s.e.m. ND, not detectable; ns, not significant.

C3H/OuJ mice have wild-type TLR4, whereas C3H/HeJ mice have a mutation in TLR4, rendering it non-functional (Poltorak et al., 1998). When these mice were given LPS, only C3H/OuJ mice with functional TLR4 showed an increase in the albumin:creatinine ratio and CD80-uria, not C3H/HeJ mice (Fig. 3A,B). The specificity of this difference was further confirmed by the observation that the TLR3 ligand poly(I:C) induced comparable albumin:creatinine ratios in both strains (Fig. 3A). To examine whether TLR4 expression on hematopoietic or non-hematopoietic cells is required for expression of CD80, we made homologous and heterologous BM chimeras between C3H/OuJ and C3H/HeJ mice using the strategy described above. Injection of LPS 8-10 weeks after irradiation led to a higher albumin:creatinine ratio in C3H/OuJ-to-C3H/HeJ chimeric mice but not in C3H/HeJ-to-C3H/OuJ chimeric mice (Fig. 3C). Homologous chimeric mice showed albuminuria similar to that seen in unmanipulated mice (Fig. S3B). CD80-uria was not observed in homologous C3H/HeJ-to-C3H/HeJ chimeras or in C3H/HeJ-to-C3H/OuJ chimeras (Fig. 3D); however, when BM from C3H/OuJ mice was used for reconstitution regardless of the recipient strain, mice developed CD80-uria (Fig. 3D). These data indicate that LPS-mediated TLR4 activation of BM-derived cells is an essential event. TLR4 expression on renal cells, such as podocytes, is not sufficient for direct LPS-mediated induction of CD80 expression and enhancement of the albumin:creatinine ratio.

We next examined the role for the major signalling intermediate in the TLR pathway, MyD88, in TLR-mediated albuminuria. LPS injection did not lead to an enhanced albumin:creatinine ratio in *MyD88*^−/−^ mice, whereas treatment with poly(I:C) did (Fig. 3E). Furthermore, LPS did not cause CD80-uria in *MyD88*^−/−^ mice (Fig. 3F). Because LPS-mediated TLR4 signalling can use both MyD88-dependent and MyD88-independent pathways, but poly(I:C) signalling via TLR3 is MyD88-independent (Lu et al., 2008; Takeda and Akira, 2004; Yamamoto et al., 2003), these data suggest that the MyD88-independent signalling pathway is sufficient to induce albuminuria upon TLR3, but not TLR4, ligation. Homologous and heterologous BM chimeras generated between B6 and *MyD88*^−/−^ mice confirmed that MyD88 expression is required in BM-derived cells and is dispensable in non-hematopoietic renal cells for LPS-mediated albuminuria and CD80-uria (Fig. S3C,D).

### Role of systemic cytokines in albuminuria and CD80 upregulation

**Fig. 4.**
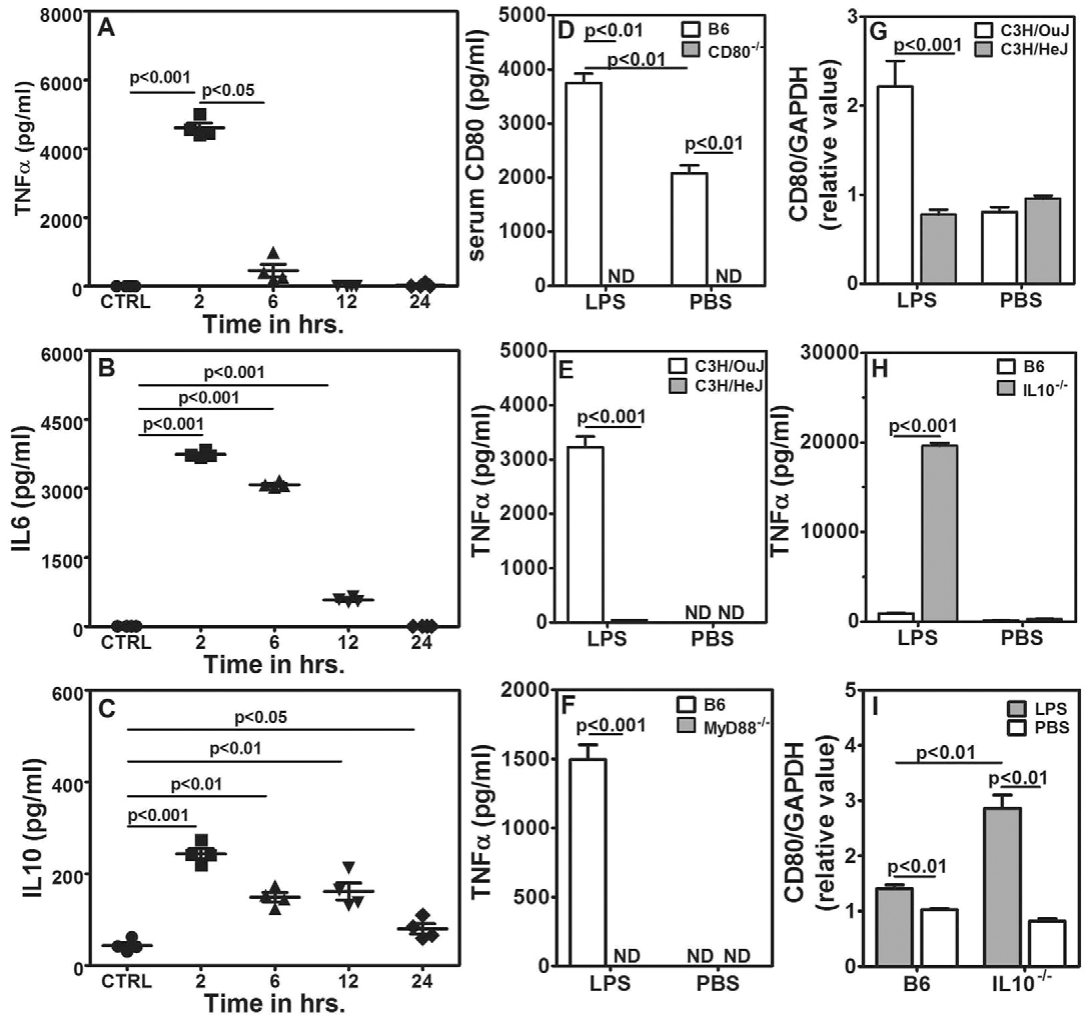
**Systemic TNFα is responsible for albuminuria and CD80 upregulation.** (A-C) TNFα (A), IL-6 (B) and IL-10 (C) levels in sera from B6 mice given PBS (CTRL) or LPS. Sera were collected at the times after treatment as indicated (four mice per group). (D) serum CD80 levels in B6 and *CD80*^−/−^ mice treated with LPS or PBS (five mice per group). (E) Serum TNFα levels 2 h after injection of LPS or PBS from C3H/HeJ and C3H/OuJ mice (five mice per group). (F) Serum TNFα levels 2 h after injection of LPS or PBS from *MyD88*^−/−^ and B6 mice (five mice per group). (G) Relative *CD80* mRNA expression levels in E11 podocytes that had been exposed to serum from C3H/HeJ and C3H/OuJ mice given PBS or LPS (*n*=8, four independent experiments). (H) Serum TNFα levels 2 h after injection of 3 μg LPS or PBS from B6 and *IL-10*^−/−^ mice (five mice per group). (I) Relative *CD80* mRNA expression levels in E11 podocytes that had been exposed to serum from B6 and *IL-10*^−/−^ mice given PBS or 3 μg LPS (*n*=6, three independent experiments). Data represents mean±s.e.m. ND, not detectable.

Because TLR expression in non-BM-origin cells was redundant for CD80-uria and alterations in the albumin:creatinine ratio, we tested the possibility that serum components derived from LPS-activated cells of BM origin are responsible for renal dysfunction leading to CD80-uria and albuminuria. TLR4-mediated macrophage activation leads to pro-inflammatory cytokine production as well as to IL-10-mediated feedback control (Inoue et al., 2014; Martinez and Gordon, 2014; Zhou et al., 2014); therefore, we measured serum levels of the pro-inflammatory cytokines TNFα and IL-6 as well as of the anti-inflammatory cytokine IL-10. Although serum levels for TNFα, IL-6 and IL-10 all peaked at 2 h after LPS injection, TNFα levels fell rapidly by 6 h, IL-6 levels more slowly by 12 h, and IL-10 levels remained detectable even at 24 h (Fig. 4A-C). Injection of LPS also led to upregulation of serum CD80 levels in B6 mice, whereas serum CD80 remained undetectable in *CD80*^−/−^ mice irrespective of LPS treatment (Fig. 4D). LPS-treated *CD80*^−/−^ mice showed an increase in TNFα levels, whereas IL-10 levels were similar to those in B6 mice (Fig. S3E,F). This is also seen in BM chimeric mice, regardless of presence or absence of CD80 expression (Fig. S3G,H). As expected, neither TLR4-mutant C3H/HeJ nor *MyD88*^−/−^ mice showed any detectable levels of TNFα in response to LPS (Fig. 4E,F), confirming previous data (Cunningham et al., 2004). Furthermore, serum from LPS-treated C3H/HeJ mice did not induce *CD80* mRNA in E11 cells, whereas serum from LPS-treated wild-type C3H/OuJ mice did (Fig. 4G), even though LPS would be expected to be present in both to the same extent.

IL-10 is an anti-inflammatory cytokine with a dominant role in alternate activation of macrophages (Martinez and Gordon, 2014; Zhou et al., 2014). *IL-10*^−/−^ mice show a hyperinflammatory phenotype. We therefore estimated serum TNFα levels in *IL-10*^−/−^ mice that had been given a lower dose (3 µg/mouse) of LPS. Sera from B6 mice exhibited relatively low levels of TNFα at this dose, whereas sera from *IL-10*^−/−^ mice showed very high levels (Fig. 4H) and these sera also induced *CD80* mRNA expression in E11 cells (Fig. 4I).

### Signalling pathway downstream of TNFα in podocytes

**Fig. 5.**
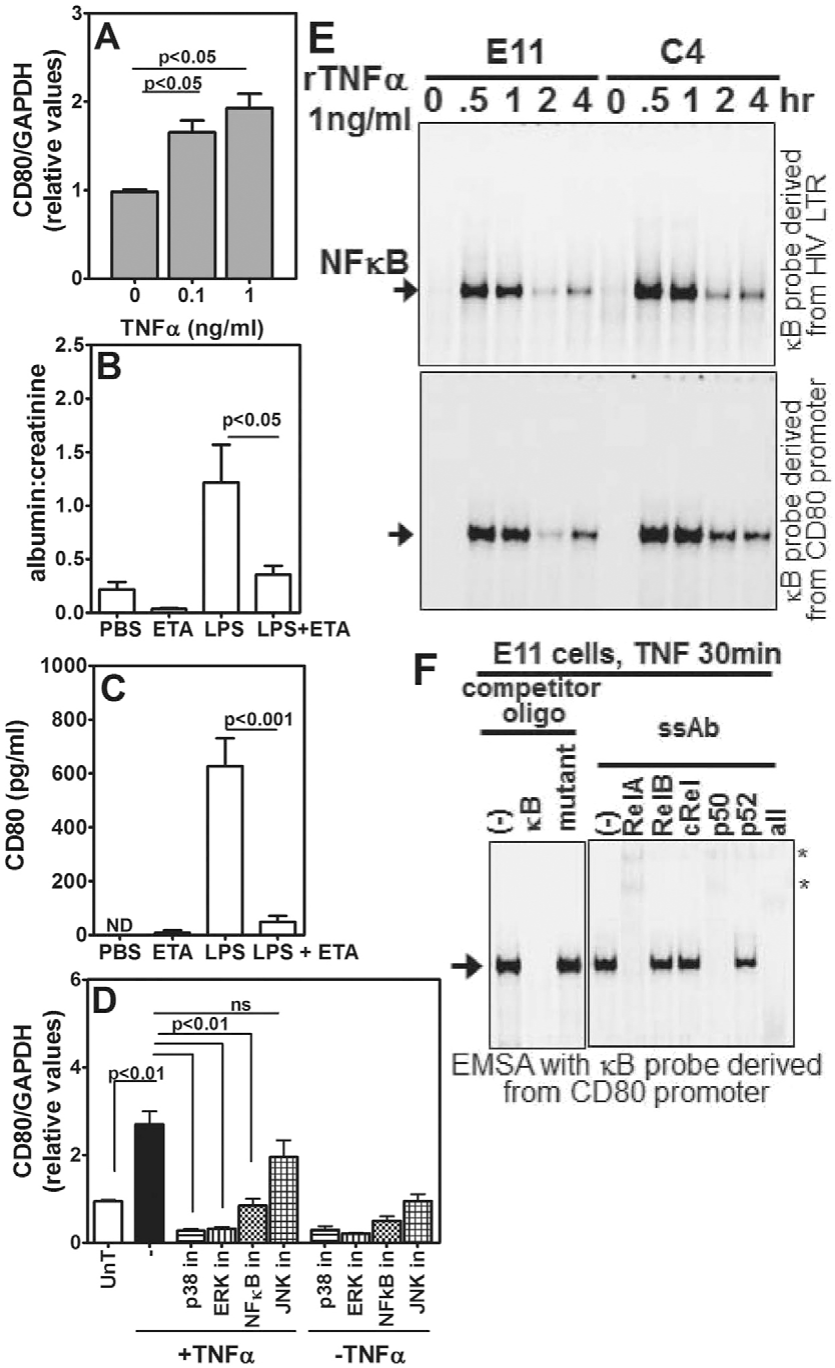
**Characterization of the role of TNFα-mediated signalling *in vivo* and *in vitro*.** (A) Relative *CD80* mRNA expression in E11 podocytes in response to increasing concentrations of recombinant TNFα (rTNFα) (*n*=8, all groups, four independent experiments). (B) Comparison of urinary albumin:creatinine ratios in B6 mice given PBS, Etanercept (ETA) alone, LPS alone or in combination as shown (five mice per group). (C) Comparison of urinary CD80 levels in mice given PBS, ETA alone, LPS alone or in combination (number of mice in each group, PBS=5, ETA=5, LPS=7, ETA+LPS=7). (D) Relative *CD80* mRNA expression levels in E11 podocytes with or without treatment with recombinant TNFα, in the presence or absence of inhibitors as shown (*n*=6, three independent experiments). Data represents mean±s.e.m. in, inhibitor; ND, not detectable; ns, not significant; UnT, untreated. (E) TNFα-mediated NFκB activation in nuclear extracts of cultured podocytes over time, determined with EMSA analysis using a DNA probe derived from the HIV LTR harbouring consensus κB sites (top panel), or from the *CD80* promoter encompassing the −2969 to −2945 region (bottom panel). (F) The specificity of NFκB binding to the *CD80* promoter sequence was examined in an oligonucleotide competition assay using 50-fold molar excess of unlabelled κB oligonucleotide or mutant oligonucleotide. *non-specific bands. Data are representative of three independent experiments. ssAb, antibodies used for supershift assay.

It has been suggested that TNFα has a role in the development of NS and that treatment with a TNFα-blocking agent could be helpful in the treatment of resistant forms of NS (Bakr et al., 2003; Lama et al., 2002; Raveh et al., 2004). Therefore, we first tested the direct effect of TNFα on *CD80* mRNA levels in the podocyte cell line E11. Although levels of *CD80* mRNA started going up from 2 h, a reliable increase could be detected at 6 h (Fig. S4A) and, hence, further experiments were performed using this time point. We found that TNFα induced *CD80* mRNA expression in E11 cells in a dose-dependent fashion (Fig. 5A). We next tested whether serum TNFα was essential for induction of LPS-mediated CD80-uria and albuminuria *in vivo*. Mice were given LPS with and without Etanercept (ETA), a TNFα antagonist (Ryu et al., 2012). Etanercept treatment substantially reduced the albumin:creatinine ratio and CD80-uria in LPS-treated mice (Fig. 5B,C). Direct intraperitoneal injection of TNFα into mice also led to albuminuria and CD80-uria in B6 mice but not in *CD80*^−/−^ mice (Fig. S4B,C), further confirming TNFα as the crucial component acting on the kidney to cause dysfunction. We next examined potential signalling intermediates by which TNFα might lead to upregulation of renal CD80. E11 cells were treated with TNFα for 6 h in the presence or absence of various well-characterized chemical inhibitors of MAPK and NFκB pathways, and subsequently *CD80* mRNA levels were examined. Although inhibition of p38 MAP kinase, MEK-Erk as well as NFκB signalling severely attenuated TNFα-induced CD80 upregulation, JNK inhibitor had only a marginal effect (Fig. 5D). Furthermore, an EMSA analysis using a DNA probe harbouring the consensus κB site revealed dose-dependent NFκB activation by TNFα in the podocyte cell line E11 (top panel, Fig. 5E and Fig. S4D), and the dynamics of NFκB activation matched TNFα-induced *CD80* mRNA expression in these cells. Supershift analyses further confirmed the participation of canonical NFκB signalling, which led to nuclear activation of NFκB dimers containing RelA-p50, with a minor contribution from RelA-p52-containing NFκB dimers (Fig. S4E). Interestingly, the *CD80* promoter possesses a binding site for NFκB in the −2969 to −2945 region upstream of the transcription start site (Zhao et al., 1996). Using a DNA probe derived from the *CD80* promoter in our EMSA, we could confirm that TNFα-induced NFκB dimers are capable of directly binding to the *CD80* promoter in podocytes (bottom panel, Fig. 5E). Supershift analyses established the participation of canonical RelA-p50 NFκB dimers in podocytes because these dimers specifically bound to the *CD80* promoter sequence (Fig. 5F). Curiously, TNFα treatment led to further augmented NFκB activation in the C4 cell line (see Materials and Methods), a derivative of an E11 cell line that stably overexpresses CD80 from an ectopic gene copy (top and bottom panels, Fig. 5E). Taken together, these data suggest that it is plausible that engagement of a positive-feedback loop under the inflammatory settings culminates in perpetuating renal CD80 expression during NS.

### Chitohexaose blocks inhibition of CD80-uria and albuminuria

**Fig. 6.**
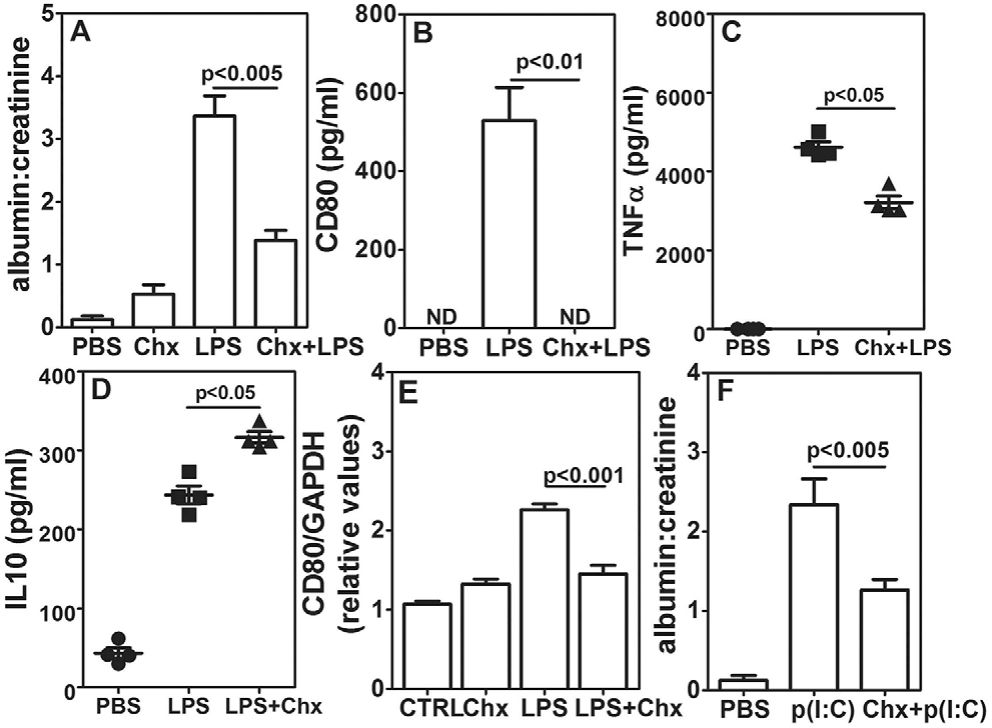
**Chitohexaose blocks TLR-mediated TNFα induction leading to inhibition of CD80-uria and albuminuria.** (A) Comparison of urinary albumin:creatinine ratios in B6 mice receiving PBS, chitohexaose (Chx), LPS or chitohexaose and LPS (five mice per group). (B) Comparison of urinary CD80 levels in B6 mice receiving PBS, LPS or chitohexaose and LPS (nine mice per group). (C,D) Serum TNFα (C) and IL-10 (D) levels in B6 mice given PBS alone, LPS alone or LPS and chitohexaose (four mice per group), 2 h post injection. (E) Relative *CD80* mRNA expression in E11 podocytes treated with sera from B6 mice exposed to PBS (CTRL), chitohexaose alone, LPS alone or LPS and chitohexaose (*n*=8, four independent experiments). (F) Comparison of urinary albumin:creatinine ratios in B6 mice receiving PBS, p(I:C) or chitohexaose and p(I:C) (five mice per group). Data represent mean±s.e.m. ND, not detectable.

We have reported that chitohexaose, a TLR4 ligand, can inhibit LPS-mediated production of pro-inflammatory cytokines *in vitro* and *in vivo*, while inducing IL-10 production from mouse and human monocyte and/or macrophages (Panda et al., 2012). We examined the effect of chitohexaose on LPS-mediated albuminuria. Mice that had been treated with chitohexaose alone showed a marginal increase in the albumin:creatinine ratio (Fig. 6A). Injection of chitohexaose just prior to LPS partially inhibited the enhancement in the albumin:creatinine ratio that was observed with injection of LPS alone (Fig. 6A). In B6 mice receiving chitohexaose and LPS, there was no detectable CD80-uria (Fig. 6B). Serum TNFα levels at 2 h post LPS treatment were significantly lower in the LPS+chitohexaose-treated group but serum IL-10 levels were higher (Fig. 6C,D). *CD80* induction in E11 cells by serum from LPS+chitohexaose-treated B6 mice was also lower (Fig. 6E). Treatment of B6 mice with chitohexaose prior to poly(I:C) injection also resulted in a lower albumin:creatinine ratio as compared with poly(I:C) alone (Fig. 6F), suggesting that despite chitohexaose and poly(I:C) using different TLRs, there might be a common pathway to inhibit albuminuria. Next, we specifically examined whether chitohexaose functioned through anti-inflammatory signalling by acting on TLR4. TLR4-responsive C3H/OuJ mice that had been given chitohexaose with poly(I:C) showed reduction in albuminuria, whereas C3H/HeJ mice were resistant to the effect of chitohexaose (Fig. S4F), confirming the requirement of functional TLR4 for chitohexaose action. We next examined whether this chitohexaose-mediated signalling required MyD88. Both B6 and *MyD88*^−/−^ mice showed similar chitohexaose-mediated inhibition of albuminuria (Fig. S4G), indicating that chitohexaose-mediated anti-inflammatory signalling through TLR is MyD88-independent.

**Fig. 7.**
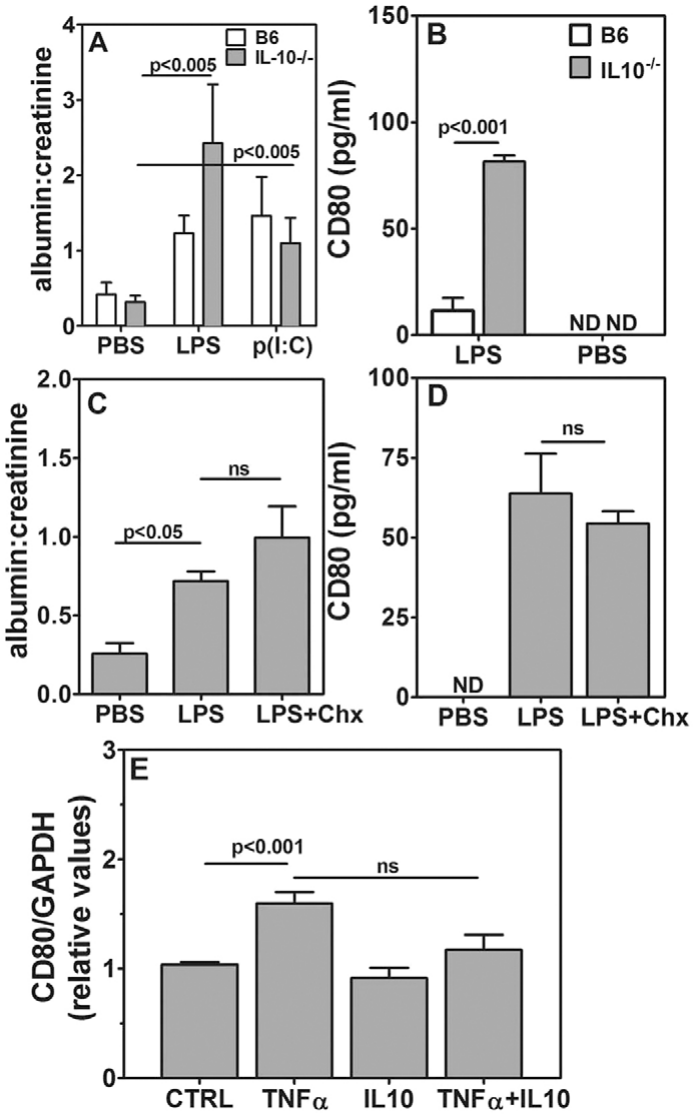
**Chitohexaose blocks TLR-mediated TNFα induction through IL-10.** (A) Comparison of urinary albumin:creatinine ratios in B6 and *IL-10*^−/−^ mice given 1 μg of LPS, 10 μg of p(I:C) or PBS (five mice per group). (B) Comparison of urinary CD80 levels in B6 and *IL-10*^−/−^ mice receiving 1 μg of LPS or PBS (six mice per group). (C) Comparison of urinary albumin:creatinine ratios in *IL-10*^−/−^ mice given PBS, 1 μg of LPS without or with 250 μg chitohexaose (five mice per group). (D) Comparison of urinary CD80 levels in *IL-10*^−/−^ mice given PBS, 1 μg of LPS without or with 250 μg of chitohexaose (five mice per group). (E) Relative *CD80* mRNA expression levels in E11 podocytes that had been exposed to PBS (CTRL), TNFα alone (1 ng/ml), IL-10 alone (50 ng/ml) or in combination (*n*=6, three independent experiments). Data represent mean±s.e.m. ND, not detectable; ns, not significant.

Because chitohexaose enhanced IL-10 levels in LPS-treated mice, we examined whether chitohexaose-mediated inhibition of albuminuria was IL-10 dependent. *IL-10*^−/−^ mice were hyper-responsive to induction of TLR-mediated CD80-uria and albuminuria, and lower doses of LPS and poly(I:C) were used for the experiments. At these doses, *IL-10*^−/−^ mice showed a higher albumin:creatinine ratio as compared to that upon PBS injection (Fig. 7A). Ratios in B6 mice were not significantly different from those in controls (Fig. 7A). At this lower dose, *IL-10*^−/−^ mice also showed CD80-uria (Fig. 7B) that was significantly more severe than that seen in B6 mice at this dose. Notably, chitohexaose could not block the enhancement in the albumin:creatinine ratio (Fig. 7C) or CD80-uria (Fig. 7D) in *IL-10*^−/−^ mice unlike in B6 mice (Fig. 6A,B), indicating that the effects of chitohexaose are mediated through IL-10 rather than being independent of IL-10. IL-10 could not block TNFα-mediated *CD80* mRNA induction in E11 podocyte cells (Fig. 7E), suggesting that IL-10 might be acting by decreasing TNFα production *in vivo*.

## DISCUSSION

Although TLR ligand-mediated induction of proteinuria is a well-known model for some components of NS, our data provide evidence that this experimental disease process requires expression of TLRs and their signalling intermediates, such as MyD88, only on BM-originating cells, and expression of CD80 only on non-BM-originating cells. These data also provide a role for TNFα and IL-10 as systemic pro- and anti-inflammatory mediators contributing to the induction of albuminuria, and suggest potential adjunct possibilities for small-molecule-based therapeutic interventions.

The mechanisms involved in the pathology of minimal change NS are still unclear. A variety of components have been shown to be altered in the disease (Berg et al., 1995; Borza et al., 2013; McCarthy et al., 2010; Nakayama et al., 2010; Reiser, 2011; Ryu et al., 2012; Shimada et al., 2011). However, although many of these studies show strong associations, they do not provide mechanistic insights. An essential role for CD80 has been shown in LPS-induced proteinuria using *CD80*^−/−^ mice (Reiser et al., 2004). CD80 is normally expressed on BM-derived cells, and further upregulation is seen in cases of infection and inflammation (Barrios et al., 2005; Leifeld et al., 1999). In contrast, renal tissue cells such as podocytes do not constitutively express CD80, but it can be induced by a variety of pro-inflammatory stimuli and contributes to altered cell biology (Eyre et al., 2011; Ishimoto et al., 2013a; Reiser et al., 2004; Yu et al., 2013). However, it is essential to address the possibility of redundant pathogenetic pathways involving CD80 expression in BM-derived and non-BM-derived cell lineages. Our data formally demonstrate that the presence of CD80 on BM-derived cells is not essential, but its absence from non-hematopoietic tissue results in an absence of albuminuria, even in the presence of TLR-mediated inflammatory signals.

Similarly, the LPS receptor TLR4 is obviously required for LPS-mediated induction of proteinuria (Cunningham et al., 2004; Reiser et al., 2004). However, TLR expression, constitutive or induced, is widespread in cellular lineages (Lu et al., 2008; Takeda and Akira, 2004). TLR4 expression on podocytes has been shown and argued to be relevant in mediating the effects of LPS on induction of proteinuria (Srivastava et al., 2013). Again, it is essential to address the possibility of redundant pathogenetic pathways involving TLR4 expression in various cell lineages. In contrast to those for CD80, our TLR4-related data formally demonstrate that although the presence of TLR4 on non-BM-derived cells, such as podocytes and other renal tissue cells, is not essential, absence of TLR4 from BM-derived cells results in an absence of proteinuria.

These findings identify TLR expression on BM-derived cells and CD80 expression in non-BM-derived cells as two crucial components that are responsible for proteinuria in this model, plausibly implicating a soluble factor that links the two. Our data identify TNFα, a pro-inflammatory cytokine, as providing the link. Although TNFα is consistently linked with septic shock and its consequences, including those on the kidney, its role in NS has not been strongly implicated. However, our findings correlate with some of the clinical observations regarding the role of TNFα (Nakayama et al., 2010; Raveh et al., 2004; Suranyi et al., 1993). Relapse in minimal change NS is associated with infections (Yap et al., 2001), and serum IL-1β, IL-6 and TNFα levels are higher during the relapse phase (Rizk et al., 2005). In some NS cases, during the relapse phase, proteinuria correlates with higher TNFα levels in the serum (Koriakova et al., 2006). Monocytes from individuals during relapse produce TNFα, which is in contrast to those during remission (Bakr et al., 2003). Thus, TNFα could be a contributor to the pathology, especially during the relapse phase of NS. Although our data provide a necessary link, there are some limitations. LPS-mediated albuminuria is one of the commonly used models for NS; however, it usually comprises microalbuminuria and is an essentially transient phenomenon. Extrapolations from such mouse models to human disease therefore require caution.

Classic or M1 activation of macrophages leads to pro-inflammatory cytokine production (Martinez and Gordon, 2014; Zhou et al., 2014) and is also observed commonly during sepsis (Clement et al., 2011; Feng et al., 2014). It has been reported that M1 activation of macrophages can lead to TNFα-mediated podocyte apoptosis, which can be blocked by using Etanercept, a TNFα antagonist (Ryu et al., 2012). Our data showing upregulation of CD80 in the podocyte cell line by recombinant TNFα, inhibition of LPS-mediated proteinuria with Etanercept and direct injection of recombinant TNFα leading to albuminuria in mice confirm that TNFα is indeed a crucial molecule that contributes to the development of albuminuria.

We have also identified some of the downstream events that occur on exposure of podocytes to TNFα. It has been previously demonstrated that RelA-p50 dimers bind to *CD80* promoter DNA in cancerous cells (Donepudi et al., 2001). Here, we show that TNFα-induced podocyte-derived RelA-p50 NFκB activity specifically recognizes the κB element that is present in the *CD80* promoter. The *CD80* promoter also harbours binding sites for AP1, which is regulated by JNK and MAPK (Donepudi et al., 2001). The ability of NFκB inhibitors, but not of JNK inhibitors, in attenuating CD80 expression suggests that AP1 is unlikely to mediate TNFα-induced CD80 expression in podocytes and that this function is dependent on NFκB activity. Our data further indicate that p38-MAPK as well as MEK-Erk signalling cooperate with the NFκB pathway in TNFα-induced *CD80* gene expression. Moreover, hyperactivation of NFκB upon TNFα treatment in cells that overexpress CD80 suggest that it is plausible that engagement of a positive-feedback loop under the inflammatory settings culminates in perpetual CD80 expression during NS. We also observed that chitohexaose on its own does not modify CD80 induction on podocytes and that its action is thus likely to be through other intermediates *in vivo*.

TLR4-mediated and CD40-mediated signals received by macrophages during early endotoxemia are known to promote *IL-10* transcription, which can in turn signal to degrade mRNA encoding TNFα (Inoue et al., 2014), thus identifying IL-10 as a crucial feedback control molecule that mediates cessation of inflammation. Our data show that, in the model system used here, IL-10 does play a similar limiting role in TLR-mediated albuminuria because *IL-10*^−/−^ mice are hypersensitive to induction of albuminuria and CD80-uria. In contrast to M1 activation, alternative or M2 activation of macrophages is seen as anti-inflammatory and is associated with IL-10 production (Martinez and Gordon, 2014; Zhou et al., 2014). We have shown previously that chitohexaose, a small molecule of nematode origin, mediates TLR4-dependent M2 activation of macrophages with prominent IL-10 production and blocks LPS-mediated endotoxemia (Panda et al., 2012). Our present data now show that chitohexaose blocks albuminuria that is induced not only by LPS but also by other TLR ligands and that chitohexaose acts at the stage of induction of pro-inflammatory cytokines from leucocytes, rather than downstream of CD80. The data also provide evidence that chitohexaose can be used as an anti-inflammatory intervention *in vivo*, regardless of the origin of the inflammatory state. Although functional TLR4 is needed for these effects of chitohexaose, the downstream signal transduction pathways do not require MyD88. Chitohexaose mediates many components of M2 activation (Panda et al., 2012), but it is the IL-10 production that is crucial for blocking TLR-mediated albuminuria, which is probably achieved by lowering TNFα concentrations *in vivo*. For the first time, our data provide evidence that chitohexaose, a small molecule, uses a global reprogramming of leucocyte activation to achieve a potential therapeutic goal.

Despite incomplete understanding of the pathophysiological mechanisms involved in minimal change NS, many therapeutic approaches have been explored, such as blocking of TNFα action with an antagonist (Raveh et al., 2004) and inhibition of the pro-inflammatory response by using an antibody to block type I interferon (Gurkan et al., 2013). Although our data suggest that chitohexaose could be another possible therapeutic option, it remains to be seen whether individuals with minimal change NS would actually benefit from its use.

Taken together, our data point to a pathway where TLR-mediated inflammation-induced TNFα secretion by BM-derived cells leads to p38 MAP kinase, MEK-Erk and NFκB signalling that are dependent CD80 induction on non-hematopoietic renal cells, resulting in proteinuria. Etanercept blocks this pathogenetic process by inhibiting TNFα-mediated CD80 induction in renal cells, and chitohexaose blocks it through TLR4-mediated M2 macrophage activation, leading to IL-10 production that negatively regulates TNFα production, thus identifying potential adjunct therapies for use in some situations of minimal change NS that have resulted from data using LPS-induced mouse albuminuria as a model system.

## MATERIALS AND METHODS

### Mice

All mice were obtained from Jackson laboratories and bred at the Small Animal Facility of National Institute of Immunology, New Delhi, India. At the initiation of experiments, all mice were 8-10 weeks old. Male mice from C57BL/6, B6.SJL, *MyD88*^−/−^, *CD80*^−/−^, *IL-10*^−/−^ mice (all H-2b background) and C3H/HeJ and C3H/OuJ (H-2k background) were used. Animals were alternately assigned to the different groups. All mouse handling was done at the National Institute of Immunology. Mice were used with the approval of the Institutional Animal Ethics Committee.

### Reagents

Mice received 100 μg LPS (Sigma, India), poly(I:C) (InvivoGen) or Pam3CSK4 (InvivoGen) with or without chitohexaose (chitohexaose, Dextra labs, UK) [250-350 μg/mouse injected 30 min prior to LPS or p(I:C)] or TNFα (3 µg/mouse) intraperitoneally. For experiments with *IL-10*^−/−^ mice, 1 or 3 μg of LPS (as specified) or 10 μg of p(I:C) was used. Etanercept (Cipla, India) (10-20 μg/mouse) was injected intraperitoneally with or without LPS. Recombinant TNFα and IL-10 (Biolegend) at the indicated concentrations were used in podocyte-cell-line-based assays.

### Preparation of BM chimeric mice

Mice were irradiated with a 9 Gy dose, and cells harvested from the BM of donor mice were given intravenously within 24 h. To create homologous and heterologous chimeric mice, BM cells from appropriate wild-type (WT) and mutant mice were transferred to both WT and mutant mice. After a minimum period of 4 weeks, recipient mice were bled to confirm reconstitution by donor marrow and for near absence of reconstitution of the endogenous population. Only those mice showing <7% of endogenous cell population were used (Fig. S1A) for assays at 8-12 weeks post radiation. Some of the mice were euthanized for further characterization of the reconstituted population, and the ability to upregulate CD80 on BM-derived cells, such as on macrophages in response to LPS. For this, splenic cells were harvested after red cell lysis, and cells were stained with anti-CD45.1 antibody conjugated to phycoerythrin (PE) (clone A20, 1:1600), anti-CD45.2 antibody conjugated to APCcy7 (clone 104, 1:800), anti-CD11b antibody conjugated to PEcy7 (clone M1/70, 1:400) and anti-CD80 antibody conjugated to FITC (clone 16-10A1, 1:200) (all from eBiosciences). Cells were analysed on a BD FACS Verse instrument (Becton and Dickinson), and data were analysed using FlowJo software (Treestar). Complete recovery from kidney injury post irradiation was ensured by checking for absence of albuminuria and CD80-uria in random urine samples at 10-12 weeks post radiation before using mice for further experiments.

### Urine collection for albumin, CD80 and creatinine detection

Spot urine was collected 24 h post treatment (unless otherwise stated) to analyse albumin, CD80 and creatinine levels. Albumin (Bethyl labs), CD80 (R&D systems) and creatinine (R&D systems) levels were detected by ELISA using commercial kits following recommended protocols. Actual values were extrapolated based on known standards run in parallel. Samples with absorbance values below detection limit in each assay were labelled as ‘not detectable’ (ND). Albumin and creatinine values calculated as µg/ml of urine were used to derive albumin:creatinine ratios.

### Collection of serum and cytokine assays

Blood was collected for separation of serum at various time points after various treatments as specified for experiments. Serum IL-6, TNFα and IL-10 levels were estimated using a protocol recommended by the manufacturer of Ready-Set-Go kits (eBioscience). Actual values for cytokines were extrapolated based on known standards. Samples with absorbance values below detection limit in each assay were labelled as ND.

### Maintenance and various assays on the E11 cell line

Murine podocyte cell line E11 was obtained from Cell Lines Service (Eppelheim, Germany). Cells were cultured on collagen (Rat tail collagen, Invitrogen)-coated culture ware and passaged in RPMI 1640 containing foetal bovine serum, antibiotics and 10 U/ml recombinant IFN-γ (Invitrogen) at 33°C. For differentiation, cells were cultured at 37°C for 10-12 days in complete medium without IFN-γ. Mouse sera collected 2 h after various treatments as indicated in each set of results were filter sterilized and added at the final concentration of 15% into differentiated E11 cultures. Recombinant cytokines, at specified concentrations, were also added into differentiated E11 cultures. After optimization of the time kinetics of TNFα effects in E11 cells, inhibitor experiments were done. For this, differentiated E11 cells were preincubated with 20 µM SB203580, 50 µM PD98059, 10 µM JNK inhibitor II or 10 µM NFκB activation inhibitor (all from Calbiochem) for 1 h prior to addition of 1 ng/ml TNFα for 6 h. Cells were harvested and lysed in Trizol (Invitrogen), and RNA was prepared as per the manufacturer's instructions.

### Detection of *CD80* mRNA in E11 cells

Total RNA (1 µg) was reverse transcribed using the Verso cDNA synthesis kit (Thermo Fisher) according to the manufacturer's instructions. Quantitative real-time PCR analysis for mouse *CD80* and *GAPDH* was performed. The primers used for the PCR were as follows: CD80f, 5′-AAGTTGTCCATCAAAGCTGACT-3′; CD80r, 5′-GAGAAGCGAGGCTTTGGGAA-3′; GAPDHf, 5′-GGGCTCATGACCACAGTCC-3′; GAPDHr, 5′-GGGATGATGTTCTGGGCAGC-3′. The results were normalized using *GAPDH* expression, and the ratio of treated to untreated serum control was calculated.

### *CD80* constructs for stable cell line C4

Mouse *CD80* was PCR amplified from a pcDNA3.1hygro+ mouse CD80 construct (a kind gift from A. Chaudhry, National Institute of Immunology) and cloned using the *Eco*RI and *Not*I sites of the pEF1 Myc His C vector in frame with the C-terminal Myc and His tags using gene-specific primers with restriction enzyme sites. The following primers were used for amplification: mCD80:249f, 5′-TACTGAATTCATGGCTTGCAATTGTCAG-3′; mCD80NotR, 5′-ATCGATTAGCGGCCGCAAGGAAGACGGTCTG-3′.

After sequence confirmation, E11 cells were transfected using Lipofectamine 2000 (Invitrogen). Stable cell lines were generated by positive selection using G418, followed by identifying single clones by using limited dilution and expanding them. One such clone, C4, consistently showed CD80 expression as confirmed by flow cytometry and western blotting, and was subsequently used for the study.

### EMSA and supershift analyses

For EMSA analyses, nuclear lysate was prepared and examined for the presence of NFκB DNA-binding activity using a radiolabelled κB site containing a DNA probe derived from the HIV LTR, as described previously (Basak et al., 2007), and from the *CD80* promoter. The sequence of DNA probe derived from the *CD80* promoter and used in EMSA was as follows (κB sites have been underlined): CD80 oligonucleotide sense strand, 5′-CTGTGGGAAAGGGGTTTTCCCAGCAGTCAGG-3′; CD80 oligonucleotide anti-sense strand, 5′-CCTGACTGCTGGGAAAACCCCTTTCCCACAG-3′.

The sequences of the κB oligonucleotide and mutant oligonucleotide used in oligonucleotide competition assay were as follows: κB oligonucleotide, 5′-CTGGGGACTTTCCAGG-3′; mutant oligonucleotide, 5′-CTGTCTACTTTCCAGG-3′. For supershift analyses, antibodies against RelA (catalogue no. sc-372, Santa Cruz), p50 and p52 (catalogue nos. BB-AB0080 and BB-AB0085, respectively, from BioBharati Ltd, India) were incubated with the nuclear extracts prior to the addition of DNA probe.

### Confocal microscopy on kidney sections

B6 and *CD80*^−/−^ mice were treated with LPS or left untreated. Twenty-four hours later, kidneys were snap frozen in liquid nitrogen with optimum cutting temperature (OCT) embedding medium (Cryomatrix, Thermo Scientific). Cryosections 3-µm thick were fixed in ice-cold methanol. After blocking with 1% BSA in PBS, primary antibodies against CD80 (R&D systems, catalogue no. AF740, 1:40) and synaptopodin (Progen, Germany, catalogue no. 61094, 1:40) were added. Sections were washed with PBS-Tween-20, followed by staining with appropriate secondary antibodies (anti-goat-Ig coupled to AlexaFluor488 for CD80 detection and anti-mouse-Ig AlexaFluor568 for synaptopodin detection, Life Technologies; catalogue nos. A11078 and A11004, respectively, at 1:500 dilution). Sections were stained with DAPI (Sigma) and mounted with 65% glycerol (Sigma). Images were taken using an Olympus FV1000 confocal laser scanning microscope. Images were acquired using a 60× objective (NA 1.42) with 2× optical zoom. AlexaFluor488 and AlexaFluor568 were excited using green and red LED lasers, respectively. Serial *z*-stack images of 0.4-µm sections were acquired. The images were processed using Olympus fluoview analysis software.

### Data analyses and statistics

Because there was no basis for expecting a pre-specified effect size, *n* was maintained as >3 on the basis of convenience and convention. No data points collected were excluded from the analyses. The data have not used any formal blinding procedures.

For statistical analysis, either Student's unpaired *t*-test (if *n*<5) or the Mann–Whitney *U*-test (for *n*≥5) were used. Values of *P*<0.05 were considered statistically significant.
